# Design, Fabrication, and Performance Test of a New Type of Soft-Robotic Gripper for Grasping

**DOI:** 10.3390/s22145221

**Published:** 2022-07-13

**Authors:** Hongjie Zhang, Wenwen Liu, Ming Yu, Yanyan Hou

**Affiliations:** 1 Tianjin Key Laboratory of Modern Mechatronics Equipment Technology, School of Mechanical Engineering, Tiangong University, Tianjin 300387, China; 2031050606@tiangong.edu.cn; 2 School of Computer and Information Engineering, Tianjin Chengjian University, Tianjin 300384, China; yuming@tcu.edu.cn; 3 School of Control and Mechanical Engineering, Tianjin Chengjian University, Tianjin 300384, China; houyanyan@tcu.edu.cn

**Keywords:** soft-robotic gripper, soft pneumatic actuator, enveloping and pinching grasp, pneumatic network structure

## Abstract

This investigation presents a novel soft-robotic pneumatic gripper that consists of three newly proposed soft actuators. The newly proposed soft actuators adopt a composite structure of two kinds of pneumatic networks which can work independently and play their respective roles in grasping. The design, analyses, and fabrication of the proposed soft actuators are introduced systematically, and then an experimental system is built to examine the output characteristics of the soft actuator. Compared with the conventional single pneumatic network-based soft actuator, the newly proposed one combines the advantages of the two pneumatic networks, and it employs a larger output force and retains desired bending deformation ability at the same time. The grasping performance test results show that the new soft gripper constituted by the proposed soft actuators has high reliability and stability whether in pinching or in enveloping grasping, and it is also competent for grasping heavier or irregular objects, demonstrating the feasibility and effectiveness of the newly proposed soft actuator, and giving it a good and wide application prospect.

## 1. Introduction

Soft robots have been receiving increased attention in many applications, such as grasping [1,2,3], manipulation [4,5], locomotion [6,7], and rehabilitation [8,9]. As an important application, grasping using the soft-robotic gripper has attracted wide interest in recent years. Compared with the rigid gripper, the soft one has excellent compliance, strong environmental adaptability, high security, and promising potential for man-machine interaction, overcoming the poor ductility and flexibility the rigid grippers usually have, as well as the limit for the grasping of fragile objects and living things. At present, some fundamental issues regarding the soft-robotic grippers have become hotspots, including design principles, kinematic modeling, bending angle and output force modeling, and control methodologies [9,10,11,12].

The soft actuator is the most important and basic component of the soft-robotic gripper, and its driving methods mainly include dielectric elastomer [13], shape memory alloy [14,15,16], tendon [17,18], and pneumatic, among which the pneumatic driving method has developed into the dominant form of soft actuators relying on its high power-to-mass ratio, high versatility, low cost, and simple control. Commonly, output force and bending angle are two critical indicators of soft actuators and they are closely correlated to the structure of the actuators. There are several typical structural forms, involving McKibben pneumatic muscle [19,20], fiber-reinforced actuators [21], and pneumatic network actuators (PneuNets) [22,23]. Compared with the first two forms, the PneuNets are easier to fabricate, and they also have the advantages of bidirectional bending and a short response time. However, owing to the adoption of soft and elastic material, the PneuNets often do not have enough pressure-bearing capacity and rigidity, resulting in their output force being small. When used to constitute a soft gripper, the PneuNets will not provide sufficient output force, causing instability and unreliability of grasping.

To improve the output force of the PneuNets for the soft gripper, many attempts have been made. These attempts can be summarized into two categories. One category aims to increase the contact area between the actuator and the grasped objects by adjusting the chamber shape, size, number, and wall thickness of the PneuNets. It is easy to understand that a bigger contact area can help to generate a larger friction force, and no doubt it is conducive to grasping heavier objects. Some other interesting attempts for increasing the contact area were also reported. For examples, Glick et al. utilized a gecko-inspired adhesive to increase the contact area and improve the output force of the soft actuator [24]. However, the preparation of the gecko adhesive is complicated and costly. Antonelli et al. designed a pneumatic actuator that was made of an inner hyper-elastic tube wrapped in an inextensible gauze with cuts, and it can be commercially available in different sizes and lengths. The most prominent characteristic of this novel actuator is that it can kinematically mirror the shape of the object to be grasped, presenting an important engineering value [25,26]. The other kind of method is to introduce some other structures or materials into the PneuNets to improve their stiffness. Park et al. inserted rigid components into the PneuNet, which was proven to be effective in increasing the output force [27]. Jiang et al. combined soft pneumatic actuators and a chain-like granular jamming mechanism to improve the output force [28]. By placing an inelastic nylon tendon, Hao et al. could adjust the effective length of the actuator for the grasping of objects with different diameters [29]. Although the granular jamming structure, fiber, and nylon can enhance the output force and improve the grasping performance of the soft-robotic grippers significantly, it limits the bending deformation of the PneuNets and increases their quality.

To improve the output force of the soft PneuNets and reduce the loss of the bending angle as much as possible, in this investigation, a novel soft pneumatic actuator was designed, based on which a new type of three-finger soft-robotic gripper was assembled. The newly proposed soft pneumatic actuator adopts a dual-module composite structure that combines two kinds of pneumatic networks. Since two pneumatic modules of the actuator can be inflated by different proportional pressure regulators in practical use, it enables them to work independently, providing their respective advantages in grasping. The output characteristics test results show that the proposed soft actuator has a larger output force compared with the traditional single pneumatic network-based soft actuator and maintains a desired bending deformation ability at the same time. The grasping performance test results show the soft gripper constituted by the proposed soft actuators presents high reliability and stability whether in pinching grasping (PG) or in enveloping grasping (EG), and it is competent for the grasping of heavier or irregular objects, presenting a good application prospect.

## 2. The Newly Proposed Soft Pneumatic Actuator

**Figure 1:**
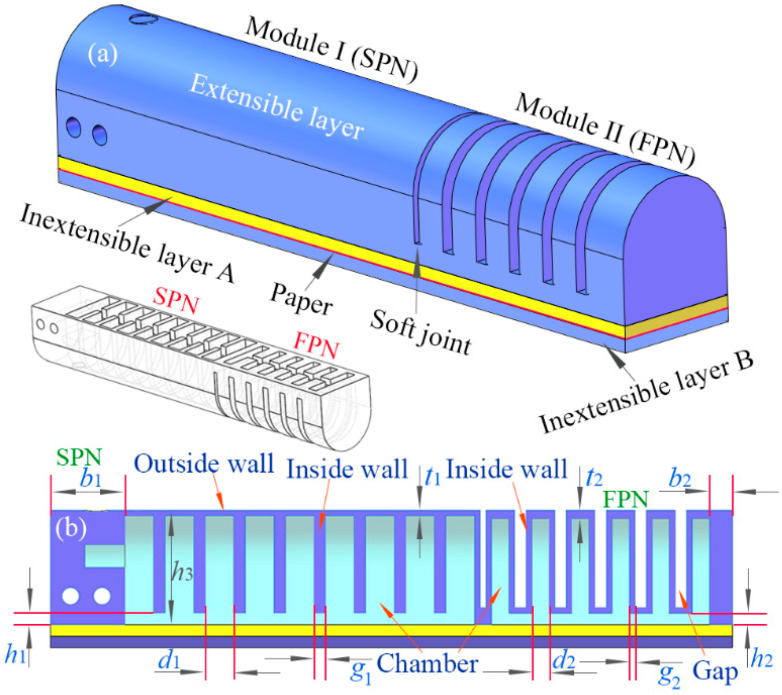
The schematic diagram for the structure of the newly proposed soft pneumatic actuator: (**a**) the structural component of the soft actuator and (**b**) a cutaway view and the relevant geometric parameters.

The structure of the newly proposed soft actuator is illustrated schematically in Figure 1a. Different from the conventional soft pneumatic actuator which often has only a single pneumatic network form, the proposed one consists of two typical pneumatic networks (extensible layers) connecting in series through two inextensible layers with an inextensible paper inserted in between. The main body of the soft actuator has a total length of 119 mm and a width of 20 mm. A sectional view of the main body is shown in Figure 1b, and the internal structure and chamber distribution can be seen visually. Moreover, the primary structural parameters are labeled as well.

The pneumatic module I at the root of the actuator adopts a slow pneumatic network (SPN), while the module II at the tip adopts a fast pneumatic network (FPN). The two pneumatic networks were named by Mosadegh et al. [30,31,32]. Due to having different structures, the two pneumatic modules present different deformation behaviors. The extensible layer of an SPN contains a series of chambers connected via a single channel. When inflated, the SPN will preferentially expand the outside wall and stretch the inside walls since the outside wall has a smaller thickness than those of the inside walls between the neighboring chambers. The extensible layer of an FPN involves a series of gaps between the adjacent chambers (as shown in Figure 1). Because the thickness of the inside wall is smaller relative to those of other walls, the inside wall is easier to expand when the FPN is pressurized, making the expanded inside walls squeeze each other. Compared with the FPN, the SPN requires a large change in volume to reach complete actuation; therefore, it needs a longer duration. In contrast, the FPN can reduce the variation in the volume required for complete actuation; therefore, it can increase the rate of actuation. These are the reasons for using the terms “fast” and “slow” to name the pneumatic networks.

Usually, the SPN can withstand a larger pressure, allowing it to output a bigger force, which is conducive to grasping larger and heavier objects and maintaining the reliability of the grasping at the same time. The FPN possesses better deformation potential, enabling it to generate a larger bending angle, which is suitable for a larger contact area. Since the two kinds of network structures have different motion characteristics and output properties, the combination of them can take full advantage of their respective features. In practical use, the two modules are controlled by different pressure regulators, making them work relatively independently and play different roles to achieve the desired adaptability and flexibility in the EG and PG tasks.

## 3. Design of the Proposed Soft Pneumatic Actuator

**Figure 2:**
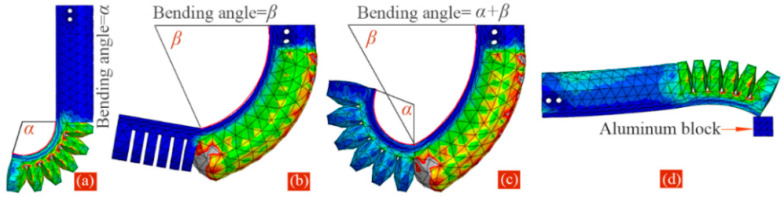
Definition of the bending angles: (**a**) module I, (**b**) module II, and (**c**) whole soft actuator. (**d**) The extraction of the output force of the actuator’s tip.

Generally, the shape, size, and chamber number of the soft actuator have significant impacts on its bending angle and output force. To compare the output properties of the proposed soft actuator under different structural parameters and determine their appropriate values, finite element analysis (FEA) was performed depending on the ABAQUS software. Since the curvature of two pneumatic modules can be thought of as approximately constant, we defined the bending angles of the two modules and the whole actuator, as shown in Figure 2a–c. To extract the bending angle information, an auxiliary means of ABAQUS software was used; a detailed extraction step can be found in Ref. [33]. To achieve the output force information, we utilized an aluminum block to contact the actuator’s tip and prevent the bending of the actuator, as shown in Figure 2d. When the soft actuator is pressurized, the contact force between the actuator’s tip and the block can be extracted, and it can reflect the tip’s output force indirectly [34,35].

### 3.1. Determination of the Chamber Number

**Figure 3:**
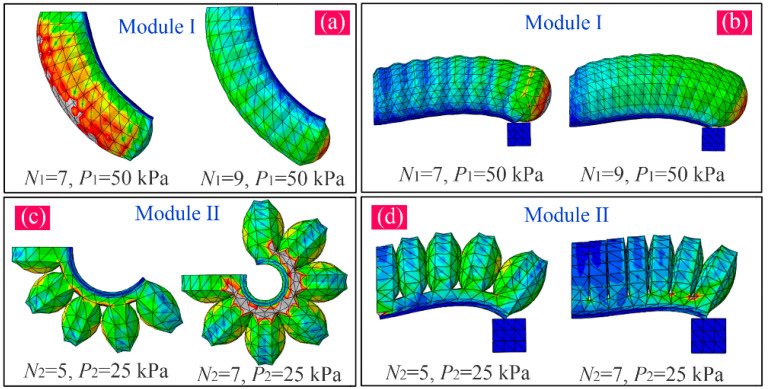
Simulation results of bending and tip force of two modules: (**a**) bending of module I when $N_{1}=7$ and $P_{1}=50$ kPa, and $N_{1}=9$ and $P_{1}=50$ kPa; (**b**) tip force of module I when $N_{1}=7$ and $P_{1}=50$ kPa, and $N_{1}=9$ and $P_{1}=50$ kPa; (**c**) bending of module II when $N_{2}=5$ and $P_{2}=25$ kPa, and $N_{2}=7$ and $P_{2}=25$ kPa; and (**d**) tip force of module II when $N_{2}=5$ and $P_{2}=25$ kPa, and $N_{2}=7$ and $P_{2}=25$ kPa.

To investigate the impact that the chamber number has on the bending angle and output force, the FE models of the two PneuNet modules were built up in software. The pressure is defined on the internal surfaces of the chambers. The Young’s Modulus of the paper used in the inextensible layer is 6.5 Gpa, and the Poisson’s Ratio is 0.2. The proposed actuator is made of Dragon Skin 30 silicone rubber, which is widely used in soft PneuNets. According to the relevant literature, the material constant C_10_ is equal to 0.11, and C_20_ and C_30_ are both equal to 0.02 when the Yeoh hyperelastic model is used [34,36,37]. Furthermore, the contact interaction is defined between the inside walls of the adjacent chambers. For the convenience of the expression, the applied pressure of module I is labeled as $P_{1}$ and that of module II as $P_{2}$. The chamber numbers of the two modules are labeled as $N_{1}$ and $N_{2}$, respectively. During analysis, the height of the chamber was set at 22 mm, and $P_{1}$ and $P_{2}$ as 50 and 20 kPa, respectively. When $N_{1}$ varied from 7 to 10, and $N_{2}$ from 4 to 7, we observed the changes in the bending angle and output force. Figure 3a,b show the simulation results of the bending and tip force of module I when $N_{1}$ adopts 7 and 9, respectively. Figure 3c,d show the simulation results of the bending and tip force of module II when $N_{2}$ adopts 5 and 7, respectively.

**Figure 4:**
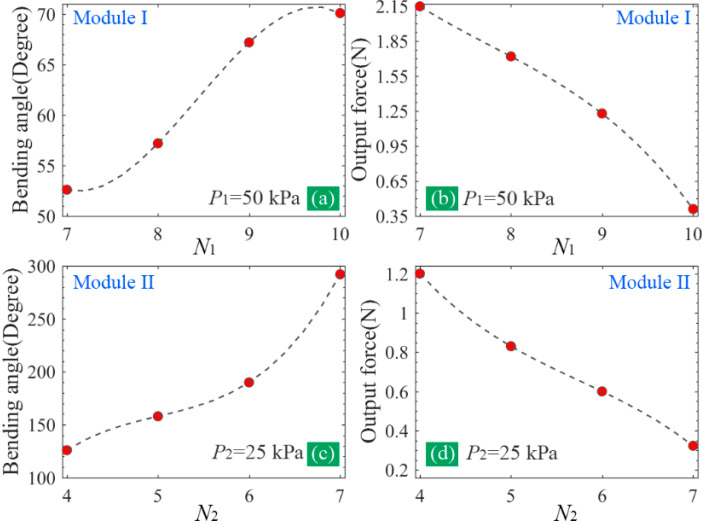
(**a**,**b**) The influence of the chamber number ($N_{1}$) on the bending angle and output force of module I and (**c**,**d**) the influence of the chamber number ($N_{2}$) on the bending angle and output force of module II.

The extracted bending angle and tip force are exhibited in Figure 4. From the trendlines in Figure 4a,b, it can be seen that the bending angle varies from 52.6° to 70.5° when $N_{1}$ changes from 7 to 10, presenting a gradually increasing tendency. However, the output force versus $N_{1}$ shows a reverse trend and it decreases from 2.15 to 0.41 N. The soft actuator is usually made of hyperelastic material; therefore, its stiffness is weaker even though the actuator is in a pressurized state. Since the increase in the chamber number will inevitably increase the soft actuator’s length, and the increase in length will further reduce the stiffness of the soft actuator, as a result, the output force will decrease. From Figure 4a, it can be seen that the relative increase in the angle becomes small when the chamber number is more than 9, indicating that the impact of the chamber number tends to weaken. The bending angle and tip force of module II as a function of the chamber number are shown in Figure 4c,d. Similar results can be seen, and the difference only lies in that the impact of the chamber number on the bending angle does not weaken with the increase in the chamber number. To ensure adequate bending and achieve as large an output force as possible, and taking into account the limitations of the total length and fabrication of the soft actuator at the same time, the chamber numbers of the pneumatic modules I and II were selected as 6 and 9, respectively.

### 3.2. Determination of the Chamber Height

**Figure 5:**
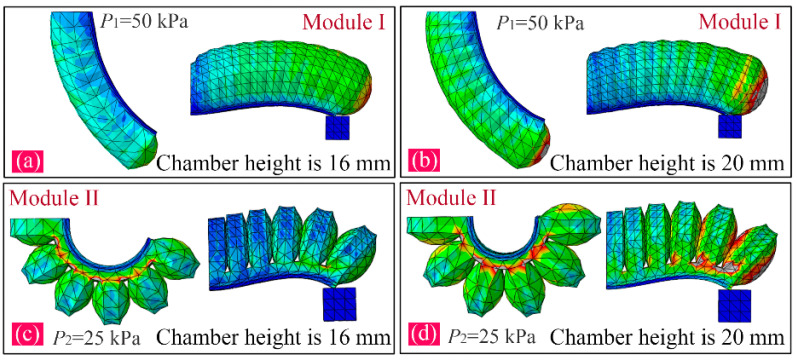
Simulation results when the chamber height adopts different values: (**a**) bending and tip force of module I at a chamber height of 16 mm, (**b**) bending and tip force of module I at a chamber height of 20 mm, (**c**) bending and tip force of module II at a chamber height of 16 mm, and (**d**) bending and tip force of module II at a chamber height of 20 mm.

Generally, the chamber height of the soft actuator can impact the volume of the chamber. When increasing within a certain range, the chamber height can improve the bending angle and the output force. However, the excessive increase in the chamber height also decreases the stiffness of the whole soft actuator, especially for the FPN-based soft actuator, which is an issue for grasping. Owing to the adoption of the different chamber structures, there is a necessity to figure out the influence that the chamber height has on the output properties of the two pneumatic modules of the proposed soft actuator, which can help us to select a suitable chamber height. For this purpose, four kinds of heights (from 16 to 22 mm with an interval of 2 mm) were considered. According to Figure 1, the chamber height is labeled as $h_{3}$. Figure 5 shows the simulation results of the bending and tip force of the two pneumatic modules at chamber heights of 16 and 20 mm.

**Figure 6:**
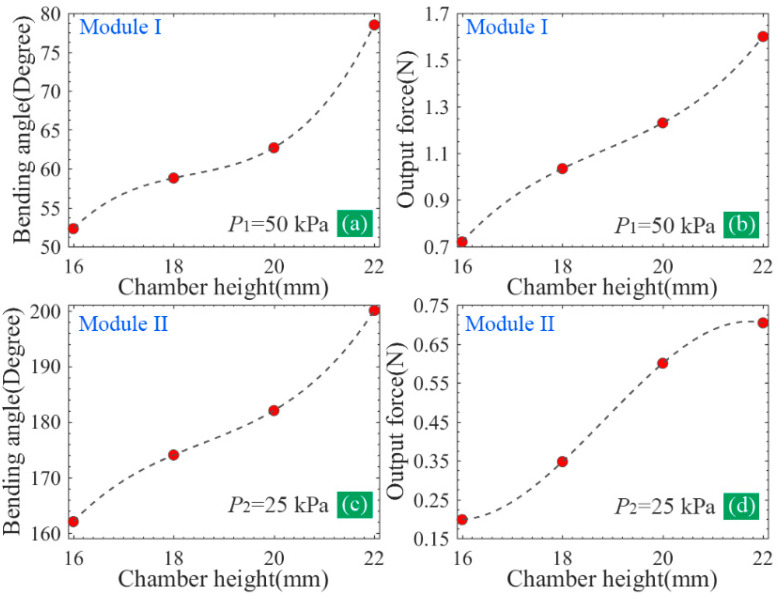
(**a**,**b**) The influence of the chamber height on the bending angle and tip force of module I, and (**c**,**d**) the influence of the chamber height on the bending angle and tip force of module II.

The variation of the bending angle and output force against the chamber height is shown in Figure 6. It can be concluded that both the bending angle and output force increase with an increased chamber height, indicating that enhancing the chamber height is conducive to the improvement of the bending and output force. Here, the chamber height of the soft actuator was determined as 22 mm. It should be noted that the influence that the chamber height has on the tip force of module II tends to be small (Figure 6d); therefore, a height of more than 22 mm was not considered. The geometric parameters of the proposed soft actuators labeled in Figure 1 are listed in Table 1.

### 3.3. Comparison of the Traditional and the Proposed Soft Actuators

**Figure 7:**
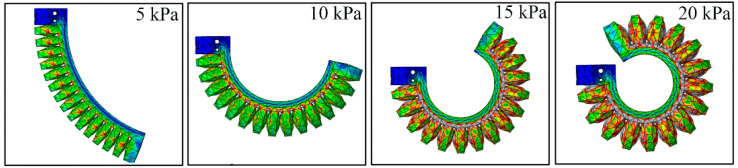
Bending deformation of the traditional soft actuator at the pressure of 5, 10, 15, and 20 kPa.

To compare the output properties of the traditional single FPN-based soft pneumatic actuator and the proposed one, a series of comparative FE simulations were performed. Before the comparison, an FE model for the conventional soft actuator was established. The two soft actuators adopted the same structural parameters, including total length, chamber number, height, and wall thickness. Considering that the maximum pressure that the two types of pneumatic networks can bear is different, the pressure applied to the traditional soft actuator and module II of the proposed actuator was limited to a range of 0–40 kPa, while that applied to module I of the proposed actuator was limited to a range of 0–80 kPa. As for why these pressure ranges were selected, we will answer that in Section 4. Figure 7 shows the bending of the traditional soft actuator at the pressures of 5, 10, 15, and 20 kPa.

**Figure 8:**
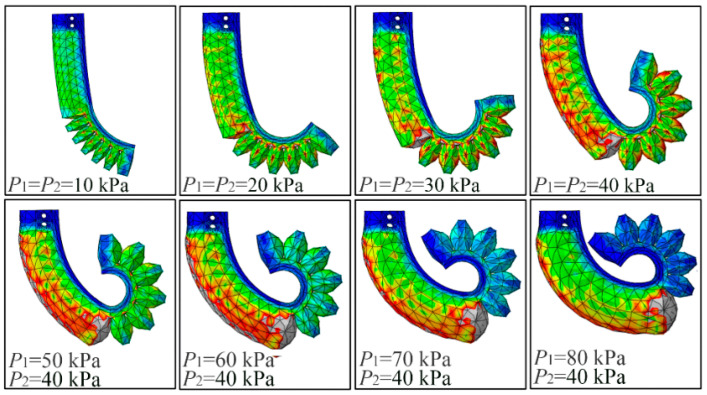
Bending deformation of the proposed soft actuator under different pressures.

**Figure 9:**
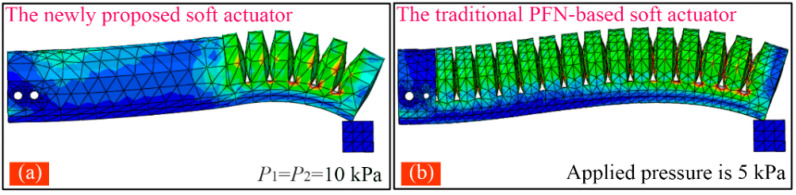
(**a**) Tip force of the newly proposed actuator when $P_{1}=P_{2}=10$ kPa, and (**b**) that of the traditional soft actuator at a pressure of 5 kPa.

Figure 8 exhibits the bending deformation of the proposed soft actuator under different pressures. It should be noted that both modules adopted the same pressure ($P_{1}=P_{2}$) as the pressure was smaller than 40 kPa. When $P_{1}>40$, $P_{2}$ remained unchanged at 40 kPa. The tip force of two kinds of soft actuators was also analyzed. Figure 9a,b show the FEA results of the traditional soft actuator under 5 kPa and the proposed actuator at 10 kPa (the two modules adopted the same pressure), respectively.

**Figure 10:**
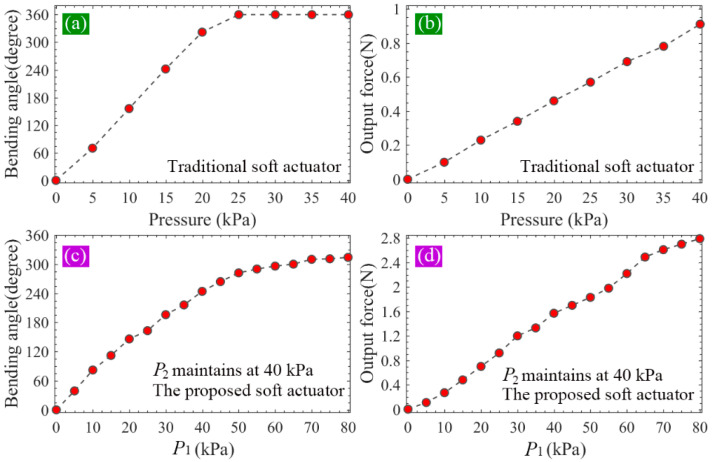
(**a**,**b**) The bending angle and output force of the traditional soft actuator under different pressures and (**c**,**d**) the bending angle and output force of the proposed soft actuator against the pressure under different pressures.

From Figure 10a,b, it can be seen the bending angle of the traditional actuator can reach 360° at the pressure of 25 kPa, and the maximum output force is 0.91 N, which appears at 40 kPa. As shown in Figure 10c,d, when the pressure applied to the two modules of the proposed soft actuator changes from 0 to 40 kPa synchronously ($P_{1}=P_{2}$), the bending angle of the whole actuator increases from 0 to 244°, and the tip force from 0 to 1.57 N. Compared with the maximum bending angle of the traditional soft actuator, that of the proposed one decreased by 32.2%. However, the maximum tip force of the proposed soft actuator increased by 72.5% compared with that of the conventional soft actuator. Considering that module I of the proposed soft actuator adopts an SPN which can bear a larger pressure than the FPN (adopted by module II) can, we continued to increase $P_{1}$ to 80 kPa and made $P_{2}$ remained at 40 kPa. It should be noted that we did not increase the pressure of the traditional actuator to 80 kPa although it can be realized in the FE simulation. This is because the traditional soft actuator cannot bear such a large pressure in practice. According to the practical test, which will be introduced in the following section, the maximum safe pressures of the fabricated FPN (used by the traditional soft actuator and module II of the proposed one) and SPN (used by module I of the proposed soft actuator) are 40 and 80 kPa, respectively. From the simulation results shown in Figure 10c,d, it can be seen, when $P_{1}$ further increases to 80 kPa, that the bending angle of the proposed soft actuator can reach 314°, and the output force can increase to 2.79 N, which is 3.07 times as big as that of the traditional actuator, presenting a large improvement.

## 4. Fabrication and Performance Test of the Proposed Soft Actuator

**Figure 11:**
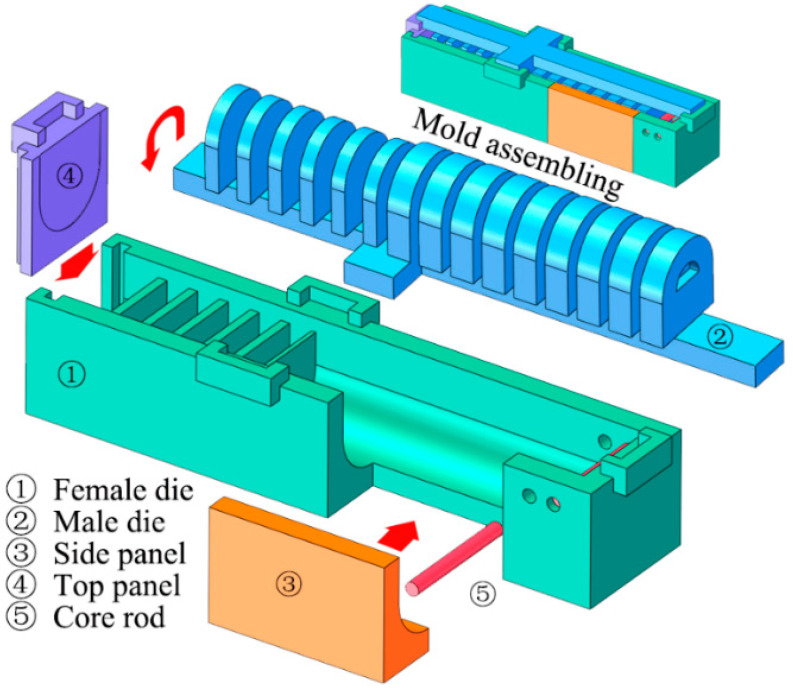
Components of the designed molds for the fabrication of the proposed soft actuator.

**Figure 12:**
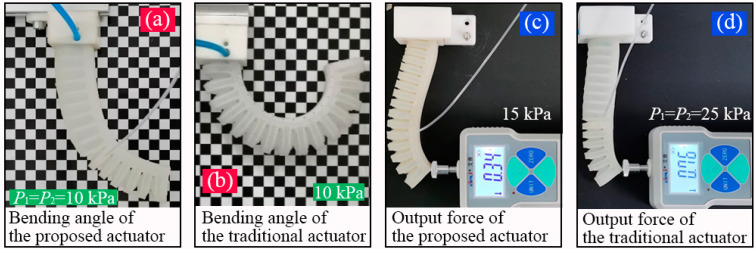
Measurement of the bending deformation and output force of two kinds of soft actuators: (**a**,**b**) bending deformation of the proposed soft actuator and the traditional one and (**c**,**d**) measuring of the output force of the proposed soft actuator and the traditional one.

To fabricate the newly proposed soft pneumatic actuator, a commercially available Dragon Skin 30 silicone rubber (Smooth-On Inc., Los Angeles, CA, USA) was used. The main body (extensible layer) and two inextensible layers were made by the integral pouring, and then these structural components were glued together to constitute a soft actuator. The molds (made by 3D printing) for the pouring are illustrated in Figure 11, in which an assembly diagram is also provided. A prototype of the proposed soft actuator is shown in Figure 12a. To compare the practical performance, a traditional FPN-based soft actuator was also fabricated, and its prototype is shown in Figure 12b.

Before the performance test and comparison, the pressure-bearing capacity and the maximum safe pressure of the two kinds of soft actuators were necessary to measure beforehand. To do this, two proportional pressure regulators (VPPM-6L, Festo Ltd., Frankfurt, Germany) were used to adjust the input pressure of the soft actuators and they had an output range of 0–600 kPa with a linearity error of ±1% FS. Four proposed soft actuators and four traditional ones were used. During the tests, when the applied pressure was 42.8 kPa, two traditional soft actuators leaked at the joint between the main body and the inextensible layer, and one burst. Something similar happened during the inflation trials of module II of the proposed soft actuator. Module I of the proposed soft actuators could work well when its applied pressure was smaller than 83.5 kPa; however, there was also an air leakage at the glued juncture when a larger pressure was used. Based on the pressure-bearing trials, we determined the maximum safe pressures of the two soft actuators: 40 kPa for the traditional soft actuator, as well module II of the proposed actuator, and 80 kPa for the module I of the proposed soft actuator.

Two groups of comparative trials were then carried out, during which the same pressure scheme as that used in FEA was adopted, namely, the pressure of the traditional soft actuator varied from 0 to 40 kPa with an increment of 5 kPa, and so did that of module II ($P_{2}$) of the proposed soft actuator. The pressure $P_{1}$ increased from 0 to 80 kPa with an interval of 5 kPa. When both $P_{1}$ and $P_{2}$ were not larger than 40 kPa, they adopted the same value, and if $P_{1}>40$ kPa, $P_{2}$ maintained its maximum safe value (40 kPa). Figure 12a shows the bending of the proposed soft actuator when $P_{1}=P_{2}=10$ kPa, and Figure 12b shows that of the conventional soft actuator at 10 kPa.

**Figure 13:**
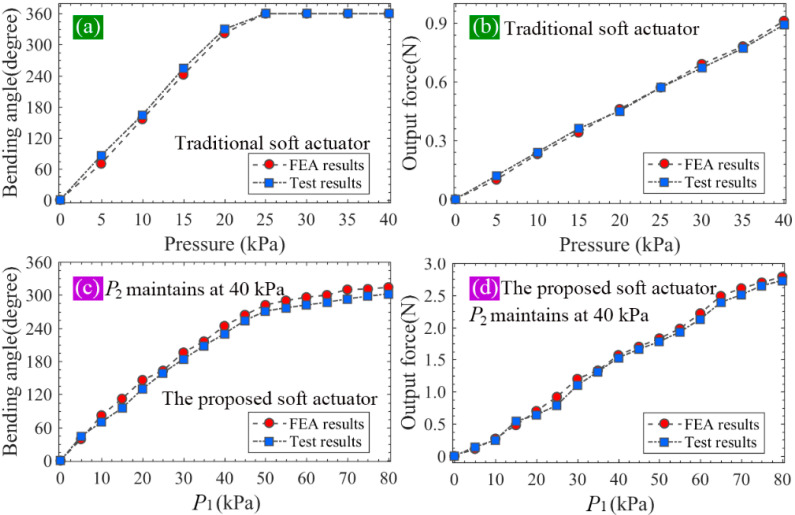
(**a**,**b**) The bending angle and output force of the traditional soft actuator under different pressures and (**c**,**d**) the bending angle and output force of the proposed soft actuator under different pressures.

To extract the bending angle of the inflated soft actuator, a special test paper was used, as shown in Figure 12a. The side length of the black and white squares was 5 mm. For each pressure or pressure combination, five inflating trials were performed. The vertical projection of the soft actuator on the test paper was used to provide the bending angle information. By utilizing a digital angle ruler (Geelii-55155) with an accuracy of 0.1°, the bending angle can be read. The average values of the measured results of five inflating trials were calculated and used as the final result. The bending angle of the traditional soft actuator versus the pressure is recorded in Figure 13a, where it is compared with that from the FEA (shown in Figure 10a). It can be seen the measured bending angle is slightly larger than the FEA result when the pressure is smaller than 25 kPa, at which point the bending angle can reach 360°. The bending angle of the proposed soft actuator is shown in Figure 13c, where it is also compared with the FFA result (shown in Figure 10c). It can be seen that the FEA results are slightly greater than those of the practical measurement, and both of them present similar tendencies with the changes in the pressure. By comparing the data in Figure 13a,c, it can be found, when $P_{1}$ is equal to $P_{2}$ and both of them are smaller than 40 kPa, that the proposed soft actuator shows a smaller bending angle than that of the traditional actuator under the same pressure level. However, when $P_{1}$ increases to the maximum safe pressure of 80 kPa and $P_{2}$ maintains at 40 kPa, the bending angle of the proposed actuator can become 300°, exhibiting an expected deformation.

To measure and compare the tip force of the two soft actuators, a high precision force gauge (AIPFI SF-10) was used. The measuring range of the gauge is 0–10 N, and the accuracy is 0.01 N. Since the output force of the soft actuator will gradually decrease from the actuator’s root to its tip, here we only focus on the comparison of the output force at the actuator’s tip. A group of measuring scenes is shown in Figure 12c (the traditional soft actuator at 15 kPa and the proposed soft actuator at 25 kPa).

Figure 13b shows the variation of the output force (average of the five inflating trials) of the traditional soft actuator against the applied pressure, where the measured results are also compared with the FEA results (shown in Figure 10b). It can be seen that both tip force curves present a similar trend with the increase in pressure. The simulated results agree with the test results well. Figure 13d records the changes of the tip force against the pressure; similarly, the measured results are compared with the FEA results as shown in Figure 10d. It can be seen that the test and FEA results are approximately linear with the input pressure, presenting a good agreement with each other. When we compare the practically measured force data in Figure 13b,d, it can be seen, under the same pressure, that the tip force of the proposed actuator is notably greater than that of the traditional one. Within 0–40 kPa, the maximum tip force of the proposed actuator is 1.53 N, which increases by 70% relative to the maximum force (0.90 N) of the traditional one. When $P_{1}$ increases to 80 kPa, the output force will reach 2.73 N, which is 3.03 times that of the traditional soft actuator, showing a significant improvement.

**Figure 14:**
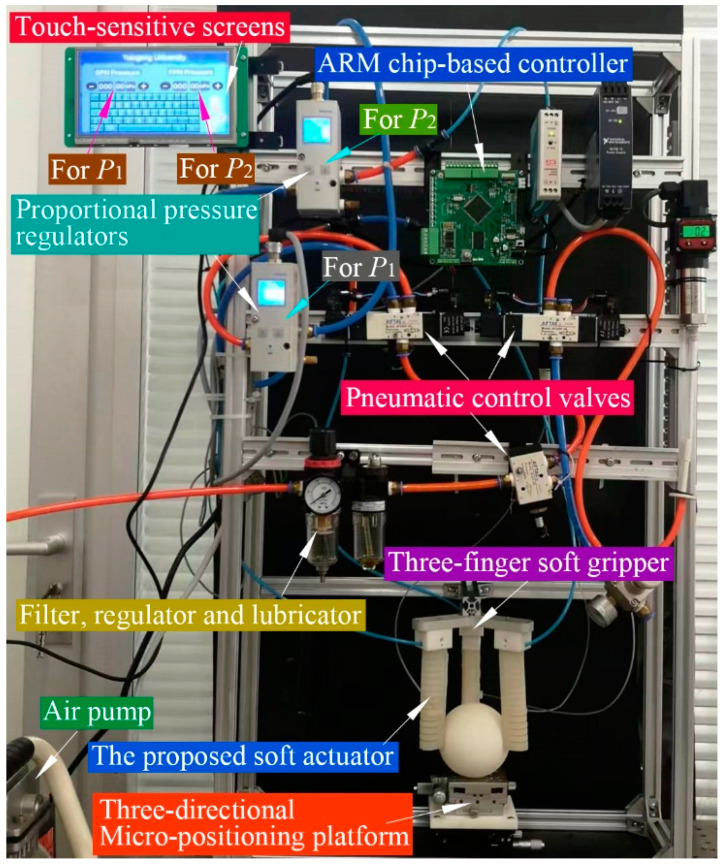
Control system of the three-finger soft gripper.

To test the grasping performance of the proposed soft actuator, a three-finger soft gripper was assembled, and an air control system was established which includes an air pump, an air treatment FRL (filter, regulator, and lubricator), several pneumatic control valves, and two proportional pressure regulators, as shown in Figure 14. To control the output pressures of the two proportional pressure regulators, a self-developed ARM microprocessor-based controller was used. Via the touch-sensitive screen of the controller, we could increase or decrease $P_{1}$ and $P_{2}$ manually to inflate the proposed soft actuators. During the grasping experiments, module I of the three soft actuators shared one proportional pressure regulator, while module II of these actuators shared another regulator, as indicated in Figure 14. Moreover, a three-directional micro-positioning platform was used to modulate the position of the object to be grasped.

**Figure 15:**
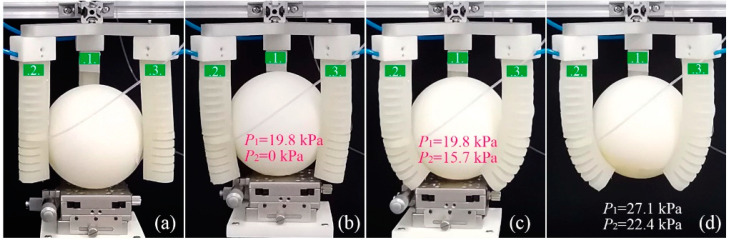
Primary stages of an EG grasping process: (**a**) the original state, (**b**) soft actuators first get in touch with the sphere, (**c**) forming an enveloping grasp with the increase in pressure, and (**d**) realization of a stable EG.

As mentioned above, the grasping mode of the soft gripper can be classified into PG mode and EG mode. For the proposed soft actuator, the PG mode can be realized in two ways: one uses the line contact or point contact between the gripper and the object, and the other is based on the plane contact. To exhibit the performance of the developed soft gripper in different grasping modes, three examples were selected. In the first example, a bigger sphere with a diameter of 10 cm was used to test the performance of the soft gripper in EG. Figure 15 records the primary stages of the grasping process. To realize the EG, we first increased $P_{1}$ to make three actuators get in touch with the sphere. When $P_{1}$ was equal to 19.8 kPa, the three soft actuators could just contact the sphere, as shown in Figure 15b. At this time, we started to increase $P_{2}$ to enable module II of the actuators to envelop the sphere. Figure 15c shows the enveloping status when $P_{1}=19.8$ kPa and $P_{2}=15.7$ kPa. To grasp the sphere stably, $P_{1}$ and $P_{2}$ continued to increase to 27.1 and 22.4 kPa, respectively. When we removed the platform, as shown in Figure 15d, we could see the soft actuator enveloping the sphere tightly, forming a larger contact area and realizing a very stable grasping.

**Figure 16:**
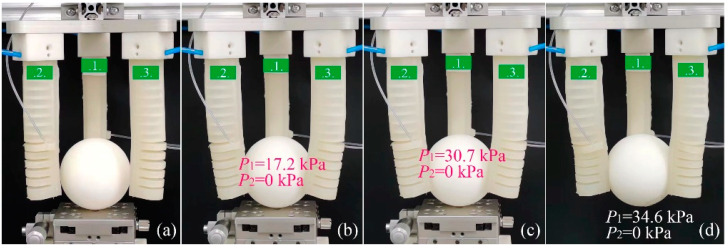
Primary stages of an EG grasping process: (**a**) the original state, (**b**) soft actuators first get in touch with the sphere, (**c**) forming an enveloping grasp with the increase in pressure, and (**d**) realization of a stable PG with a plane contact.

Figure 16 exhibits the primary stages of a PG example in which a smaller sphere was used. To realize the plane contact between the sphere and module II of the soft actuator, $P_{2}$ was kept at zero during the whole grasping process. When $P_{1}$ increased to 17.2 kPa, module II of the soft actuators could contact the sphere, as shown in Figure 16b. To produce a large enough pinching force, we continued to increase $P_{1}$ to 30.7 kPa, and the sphere was pinched gradually, as shown in Figure 16c. When $P_{1}$ increased to 34.6 kPa, we removed the platform and it could be seen that the gripper pinched the small sphere successfully, as Figure 16d shows.

**Figure 17:**
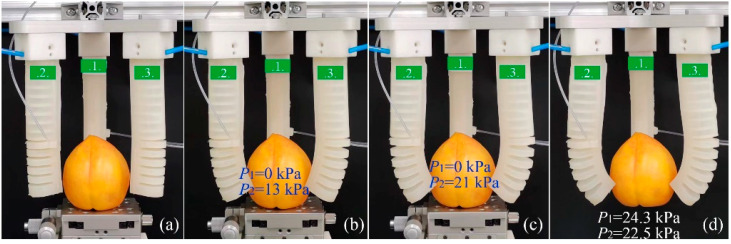
Primary stages of an EG grasping process: (**a**) the original state, (**b**) soft actuators first get in touch with the sphere, (**c**) forming an enveloping grasp with the increase in pressure, and (**d**) realization of a stable PG with line contact.

Another PG example is presented in Figure 17. In this example, a yellow peach was used and the grasping was realized by using the tips of the soft actuators. Within the grasping process, $P_{2}$ was adjusted first to make the actuator’s tip get in touch with the peach. Figure 17b shows the first contact of the soft actuator and the peach when $P_{2}=13$ kPa. With $P_{2}$ rising to 21 kPa, the peach was gradually pinched tightly by the actuator’s tips, as shown in Figure 17c. To make a larger tip force, $P_{1}$ and $P_{2}$ started to increase at the same time. As $P_{1}=24.3$ kPa and $P_{2}=22.5$ kPa, we removed the platform, and the peach was grasped stably by the gripper, as shown in Figure 17d. From the above examples, it can be seen the soft gripper constituted by the proposed soft actuator has good performance in both EG and PG. Since the proposed soft actuator adopts a composite structure of two kinds of pneumatic networks, it employs different output properties through different pressure combinations, presenting the expected flexibility and adaptability.

**Figure 18:**
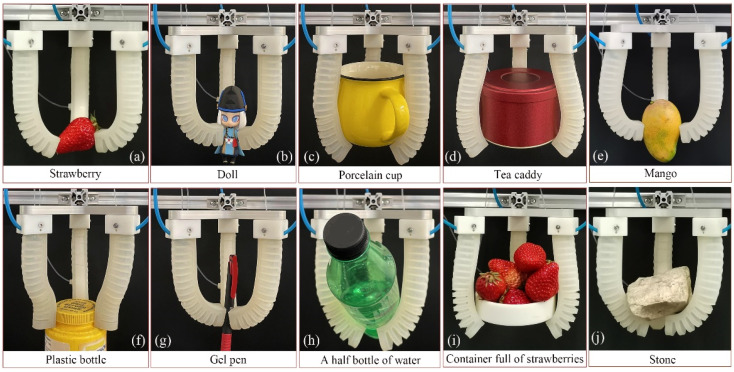
Successful grasps of (**a**) strawberry, (**b**) doll, (**c**) porcelain cup, (**d**) tea caddy, (**e**) mango, (**f**) plastic bottle, (**g**) gel pen, (**h**) half a bottle of water, (**i**) container full of strawberries, and (**j**) stone.

To further examine the grasping performance of the soft gripper, a series of objects with different shapes, sizes, and weights were used, as seen in Figure 18. During the experiments, if a grasp could remain stable for more than 60 s without dropping, it was defined as a successful grasp. It can be seen that the proposed soft gripper could grasp some small, irregular, and heavier objects in PG mode, such as a strawberry (Figure 18a), a doll (Figure 18b), a gel pen (Figure 18g), a mango (Figure 18e), and a plastic bottle (Figure 18f). It could also grasp heavier objects of a larger size in EG mode, such as a porcelain cup (Figure 18c) and a tea caddy (Figure 18d). The soft gripper could reliably grasp a 375g payload at a low input pressure ($P_{1}=P_{2}=20$ kPa), such as half a bottle of water (Figure 18h). Moreover, it could also bear a stone with a quality of 447 g, as shown in Figure 18j. The grasping performance test results show that the new soft gripper constituted by the proposed soft actuators presents high reliability and stability whether in PG mode or EG mode, and it is competent for grasping objects with different shapes, sizes, and weights, demonstrating the feasibility and effectiveness of the newly proposed soft actuator.

## 5. Conclusions

This investigation presents a novel soft-robotic pneumatic gripper for grasping. The soft gripper consists of three newly proposed dual-module soft actuators that combine two kinds of pneumatic network structures. Based on the FEA, the influence of chamber number and height on the bending deformation and output force of the soft actuators was investigated, based on which their suitable values were determined. After finishing the fabrication of the soft actuators, a series of tests, comparisons, and analyses regarding the output properties of the soft actuators were then carried out. Finally, a soft gripper was assembled and its grasping performance in PG and EG was tested, and the following conclusions were obtained:

1. Chamber number and height have significant influences on the bending angle and output force of the proposed soft actuators. With the increase in the chamber number, the bending angle of the two pneumatic modules increases accordingly, while the output force presents a reverse tendency. Within a certain range, with the increase in the chamber height, both bending angle and output force tend to increase.
2. Compared with the conventional single pneumatic network-based soft actuator, the proposed one combines the advantages of the two typical pneumatic network structures. The two modules of the proposed soft actuator can work relatively independently, which can improve the adaptability and flexibility of grasping.
3. Performance test results show the proposed soft actuator has a larger output force and an expected bending angle. Compared with the traditional single pneumatic network-based soft actuator, the output force of the proposed one increased by 70% when the two soft actuators worked at 40 kPa. When the two soft actuators worked under their respective maximum safe pressure, the output force of the proposed soft actuator could reach 3.03 times that of the traditional one, at the same time keeping an expected bending angle output, showing a significant improvement.
4. Grasping performance test results show that the new soft gripper constituted by the proposed soft actuators presents high reliability and stability whether in PG or EG, and it is competent for grasping heavier or irregular objects, demonstrating the feasibility and effectiveness of the newly proposed soft actuator, and giving it a good and wide application prospect.
5. Since the newly proposed soft actuator has two pneumatic modules, there are many possible pressure combinations to approach the object, get in touch with it, increase the force, and grasp it. Although the improvement in the structure proved meaningful, it brings a series of issues, such as how to obtain the optimal pressure combination, how to develop the relevant control algorithms, and how to make the proposed soft actuator work well in practice. To solve these problems, some basic issues must be investigated deeply, such as the analytical models of the bending angle, output force, and kinematic behavior. These issues will be studied in the future.

## Figures and Tables

**Table 1:** Parameters of the newly proposed soft pneumatic actuator.

**Parameters**	$b_{1}$	$b_{2}$	$d_{1}$	$d_{2}$	$g_{1}$	$g_{2}$
**Values (mm)**	13	4	5	3	2	1
**Parameters**	$h_{1}$	$h_{2}$	$h_{3}$	$t_{1}$	$t_{2}$	--
**Values (mm)**	2.5	2.5	22	1.5	1.5	--

## Data Availability

Not applicable.
